# Prevalence and predictors of heated tobacco products use among male ever smokers: results from a Korean longitudinal study

**DOI:** 10.1186/s12889-021-10344-4

**Published:** 2021-02-08

**Authors:** Jeeeun Yi, Cheol Min Lee, Seung-sik Hwang, Sung-il Cho

**Affiliations:** 1 Division of Public Health Science, Graduate School of Public Health, Seoul National University, 1, Gwanak-ro, Gwanak-gu, Seoul, Republic of Korea; 2 Department of Family Medicine, Healthcare System Gangnam Center, Seoul National University Hospital, 39th Fl, Gangnam Finance Center, 152, Teheran-ro, Gangnam-gu, Seoul, 06236 Republic of Korea

**Keywords:** Electronic nicotine delivery systems, Tobacco, Nicotine, Policy

## Abstract

**Background:**

This study examined sociodemographic and tobacco-related factors of heated tobacco products (HTPs) use among adult ever smokers in South Korea where the sales of HTPs have been rapidly increasing since their launch in June 2017.

**Methods:**

Before the launch of HTPs in Korea, participants comprised male ever smokers (234 current smokers and 37 quitters) who participated in the Korea National Health and Nutrition Examination Survey from 2015 to 2017 through one-to-one interview survey and agreed to participate in the follow-up surveys through telephone in December 2017. Data were analyzed using logistic regression, to explore sociodemographic and smoking behavior-related factors of HTPs use.

**Results:**

Overall, 10.7% (29/271) of participants responded to using HTPs and 8.1% (22/271) were current HTPs users at the time of the follow-up survey. Multivariate analysis showed that HTPs use is associated with middle age (36 to 49 years old) (aOR = 3.72, CI = 1.16–12.0) (vs. ≥ 50 years), higher income (4Q vs 1Q: aOR = 2.71, CI = 1.16–6.34), and higher educational level (college or higher: aOR = 2.40, CI = 0.87–6.60). Also, vaping experience at baseline was highly associated with HTPs use (aOR = 3.11, CI = 1.22–7.93 for the former experience; aOR = 9.14, CI = 2.34–35.6 for current). However, smoking amount and level of motivation for smoking cessation were not found to be predictors of future HTPs use when limited to current smokers at baseline.

**Conclusions:**

The results showed that vaping experience regardless of current smoking behavior and higher socioeconomic status were found to be associated with subsequent HTPs use among ever smokers. Further studies are required to explore whether this association is causal.

## Background

Heated tobacco products (HTPs) are new technological devices that are designed to undergo heat process instead of combusting and release nicotine and other volatile compounds into an inhalable aerosol [1]. An updated hybrid version, “IQOS” by PMI, was first launched in Japan in September 2014 [2]. PMI applied its HTPs to the U.S. Food and Drug Administration (FDA) for a modified risk tobacco product (MRTP) registration [3]. Although to prevent teenagers from accessing and exposing this new product, stringent marketing restrictions were placed on HTPs in advance, the US FDA finally approved it [4].

In South Korea, three brands of HTPs have been introduced to the market since June 2017. These products have drawn tremendous attention from smokers who are concerned with their health as well as exposing others from smoking [5]. Accordingly, the market share of HTPs reached about 10% of total tobacco sales within one year of their introduction [6]. As PMI claims that IQOS emits no smoke because HTPs do not undergo combustion, these new products can be more socially acceptable [7, 8]. However, given the fact that these new tobacco products are being sold globally, the debates on health consequences are growing markedly.

Several recent studies investigated the prevalence of HTPs use [5, 9] and their association with mental health in South Korea [10]. Also, a few cross-sectional studies have investigated the characteristics of awareness or use of HTPs [2, 5, 11–13]. However, to the best of our knowledge, no prospective studies have investigated the factors that affect adults’ susceptibility to HTPs use.

## Methods

The data of 271 male ever smokers were collected from the Korea Centers for Disease Control and Prevention (KCDC)-funded. This study was conducted in December 2017, six months after the launch of HTPs in South Korea. Participants in the research were recruited from the previous years of KNHANES baselines (2015–2017) and agreed to participate in follow-up surveys annually until 2020. Questions about using HTPs were “Have you ever used HTPs?” and “In the past 30 days have you used HTPs every day, on some days, or not at all?” [14] The baseline questionnaire included socio-demographic variables such as age, marital status, educational standards, and households. Other smoking-related characteristics such as baseline smoking (former/current), vaping status (former/current vaper (use in the past month)), number of cigarettes per day, and motivation for quitting were also considered using the transtheoretical model [15]. The baseline data were analyzed by bivariate analysis (chi-square test). After identifying variables that were significantly related to HTPs use, the multivariate logistic regression analysis was performed to examine baseline factors related to future HTPs use.

## Results

At the baseline, 234 participants responded as current smokers (86.3%) and 37 as quitters (13.7%).

At the follow-up survey, the number of current smokers has decreased to 76.8% (*n* = 208/271). More than 40 and 10% of respondents said they had e-cigarettes in the past year and HTPs in the past month. Age, household income level, and vaping status reported at the baseline survey were found to be significantly associated with future HTPs use. Several factors such as having a higher education level, and cohabitation with partners were noticeable in the bivariate analysis; however, were not statistically significant. The baseline smoking status (former or current) was also not related to future HTPs choices (*p* = 0.593).

Table 1 shows that people aged 36 to 49 were three times higher than those over 50. People with higher household income status (4Q) were three times as high as those with the lowest income. Both former and current vaping at baseline were highly related to the use of HTPs. Although those with college or higher educational attainment were more likely to use HTPs, this relationship was marginally significant (*p* = 0.09).

**Table 1:** ORs of heated tobacco products (HTPs) use among male ever smokers (*n* = 271)

		%	aOR	95% CI	*P*
Age	50+	5.1	1 (ref)		
	36–49	18.7	**3.72**	1.16–12.0	0.027
	~ 35	5.9	0.61	0.14–2.62	0.51
Education	~high school	6.8	1 (ref)		
	College or over	14.6	2.40	0.87–6.60	0.09
*Household income	1Q ~ 3Q	8.1	1 (ref)		
	4Q	21.1	**2.71**	1.16–6.34	0.021
E-cigarette use status	Never	7.3	1 (ref)		
	Former	13.6	**3.11**	1.22–7.93	0.017
	Current	27.8	**9.14**	2.34–35.6	0.001

Data are ORs (95% CI) unless indicated otherwise. *P* values were calculated after adjustment for age (categorical), education (categorial), household income (1Q, minimun to 318,700 KW; 2Q, 318,701 to 2,000,000 KW; 3Q, 2,000,001 to 3,990,000 KW; 4Q, 3,990,001 KW to maximum), and vaping status (categorical)

## Discussion

HTPs sales exceeded 5% of total cigarette sales four months after its launch and reached 10% in 11 months in South Korea [6]. At six months after IQOS was launched, 10.7% of smokers so far responded they had experience with HTPs. Although the smoking prevalence among Koreans has diminished over time, new tobacco products still have a big impact on the existing tobacco market.

Our study found that being middle-aged (36–49 years old), have higher incomes, higher educational attainments, and prior vaping experiences are associated with future HTPs use. These findings are consistent with Japan’s 2018 study which showed that current smoking, ever vaping, and higher socioeconomic status to be related with subsequent use of current HTPs [11]. The amount of smoking reflecting nicotine dependence and the motive for smoking cessation were found not to be associated with the following use of HTPs. It is presumed that smokers have chosen HTPs as alternative tobacco products because they smell less and are less unhealthy. While e-cigarette users tend to choose HTPs as an aid to quit smoking, HTPs can be irrelevant to smoking cessation efforts.

Several factors were investigated to experiment HTPs using longitudinal data, however, some limitations appeared on our study. First, our study analyzed only 271 male subjects and secondly, our results such as the prevalence of each tobacco product cannot be applied to the general population.

## Conclusions

Previous vaping experience, middle-age (36–49 years old), and higher income were found to be the major factors of future use of HTPs among study participants, regardless of smoking status at the baseline.

## Data Availability

The datasets generated and/or analysed during the current study are not publicly available due to the policy of the Korea Center for Disease Control and Prevention, which funded this study.

## Abbreviations

HTPs: Heated tobacco products
aOR: Adjusted Odds Ratio
CI: Confidence Interval
PMI: Philip Morris International
FDA: Food and Drug Administration
KCDC: Korea Centers for Disease Control and Prevention
KNHANES: Korea National Health and Nutrition Examination Survey
